# Correlation between physical symptoms and overall comfort in hospitalized cancer patients in palliative care*

**DOI:** 10.1590/1980-220X-REEUSP-2025-0511en

**Published:** 2026-07-06

**Authors:** Ana Luísa Teixeira da Costa Durante, Lívia Costa de Oliveira, Andrea dos Santos Garcia, Sônia Regina de Souza, Daniel Aragão Machado, Carlos Roberto Lyra da Silva

**Affiliations:** 1 Instituto Nacional do Câncer, Rio de Janeiro, RJ, Brazil.; 2 Universidade Federal do Rio de Janeiro, Escola de Enfermagem Anna Nery, Departamento de Enfermagem Médico-Cirúrgico, Rio de Janeiro, RJ, Brazil.; 3 Universidade Federal do Estado do Rio de Janeiro, Escola de Enfermagem Alfredo Pinto, Programa de Pós-Graduação em Enfermagem e Biociências, Rio de Janeiro, RJ, Brazil.; 4 Universidade Federal do Estado do Rio de Janeiro, Programa de Pós-Graduação em Saúde e Tecnologia no Espaço Hospitalar, Rio de Janeiro, RJ, Brazil.

**Keywords:** Palliative Care, Patient comfort, Oncology Nursing, Symptom assessment, Quality of Life

## Abstract

**Objective:**

To analyze the correlation between the intensity of physical symptoms and overall comfort levels in hospitalized cancer patients receiving palliative care.

**Method:**

This quantitative, descriptive, and analytical study was conducted with 155 patients admitted to palliative care units at a university hospital. The General Comfort Questionnaire (GCQ) and the Edmonton Symptom Assessment Scale (ESAS) were used. Data were analyzed using descriptive and inferential statistics, employing Pearson’s correlation (p ≤ 0.05).

**Results:**

The ESAS symptom assessment revealed a high symptomatic burden, with anxiety, loss of appetite, and depression standing out as the symptoms with the highest average intensity. A statistically significant negative correlation was found between the intensity of most symptoms and overall comfort, indicating that increased symptom burden was associated with reduced comfort.

**Conclusion:**

The increased intensity of physical symptoms is associated with a significant reduction in overall comfort, reinforcing the importance of effective strategies for managing these symptoms in palliative nursing practice.

## INTRODUCTION

Comfort is one of the cornerstones of nursing practice in palliative care and represents an essential indicator of the quality of care. The Comfort Theory understands comfort as a dynamic state of relief, tranquility, and transcendence in the physical, psycho-spiritual, sociocultural, and environmental dimensions. Among these, the physical dimension is the most directly affected by the presence of symptoms, which compromise well-being and the perception of quality of life^(1)^. Hospitalized cancer patients in the palliative care phase experience multiple physical symptoms, such as pain, fatigue, dyspnea, and loss of appetite, which directly interfere with their comfort and care experience^(2)^.

The progression of cancer often intensifies the presence of multiple and difficult-to-control symptoms, causing physical, emotional, and existential suffering. Managing these symptoms is a priority in palliative care, as it is directly related to the promotion of comfort and to quality of life^(2)^. In this context, the systematic measurement of symptom intensity becomes essential to guide individualized interventions and evaluate the effectiveness of nursing practices aimed at relief and well-being^(3)^.

Physical comfort represents the experience of relief and tranquility resulting from the satisfaction of basic bodily needs and the control of pain and other symptoms^(1,2,3)^. The absence of comfort translates into discomfort, fatigue, and suffering, interfering with the cancer patient’s homeostatic and emotional balance^(4)^. Therefore, comfort assessment should be integrated into care routine, allowing for the early identification of changes that negatively impact the general condition and the therapeutic response to treatment^(5)^.

Recent studies with patients in palliative care indicate a significant correlation between intense physical symptoms and low levels of overall comfort, suggesting that discomfort is a direct consequence of symptomatic accumulation^(3)^. The literature shows that the greater the intensity of pain and fatigue, the lower the scores for physical and overall comfort^(4,5,6)^. This finding reinforces the nurse’s role in the continuous monitoring of symptoms and in the application of standardized scales such as the Edmonton Symptom Assessment Scale (ESAS) and in the adoption of evidence-based practices for symptomatic control^(7,8,9)^.

The use of General Comfort Questionnaire (GCQ) in the context of palliative care allows us to understand comfort as a multidimensional phenomenon and to assess its physical, psycho-spiritual, sociocultural, and environmental dimensions in an integrated way. In hospitalized cancer patients, the QCG (Quantitative Compensation Chart) has demonstrated sensitivity in detecting variations in well-being in response to nursing interventions, making it possible to identify the factors that most compromise comfort^(3–5)^. This tool assists in formulating care plans centered on the patient’s needs, especially in managing symptoms that affect both body and mind simultaneously^(3,4,5,6,7)^.

Therefore, investigating the relationship between physical symptoms and overall comfort contributes not only to improving care practices, but also to strengthening quality indicators in palliative care. By highlighting that physical discomfort is strongly associated with symptomatic intensity, it becomes possible to direct interdisciplinary efforts towards early and effective interventions^(8,9,10,11,12)^. The integration of symptom assessment and comfort measurement should be considered an essential component of good palliative nursing practices, with a direct impact on dignity, well-being, and the humanization of care^(13)^.

Although the relationship between physical symptoms and comfort in cancer patients is already recognized in the literature, there is a scarcity of studies investigating these associations in a correlational way in hospitalized individuals receiving palliative care, especially in the Brazilian context and using validated instruments in a combined manner. Most research focuses on outpatient settings or specific stages of the disease^(7,9,14)^. Thus, the present study aimed to analyze the correlations between the intensity of physical symptoms and the levels of overall comfort in hospitalized cancer patients receiving palliative care.

## METHOD

### Study Design

This is a quantitative, observational, descriptive, and analytical study with a cross-sectional design, based on Kolcaba’s Theory of Comfort^(1)^.

The cross-sectional design allowed for data collection at a single point in time during hospitalization, facilitating the analysis of interrelationships between the symptoms assessed by ESAS^(3,15,16,17,18)^ and the comfort levels measured by the GCQ^(1,11,12)^.

Analyses of the association between overall comfort and sociodemographic variables were performed using mean comparison tests (Student’s t-test or analysis of variance – ANOVA, depending on the type of variable), while the relationships between symptom intensity and overall comfort levels were examined using Pearson’s correlation coefficient.

### Research Location

The study was conducted at Cancer Hospital IV (HC IV) of the National Cancer Institute (*INCA*), located in the city of Rio de Janeiro, Brazil. INCA is an institution linked to the Ministry of Health and constitutes the main national public reference center for cancer prevention and control, with four healthcare units distributed throughout the city: HC I, HC II, HC III and HC IV.

HC IV is the palliative care unit of *INCA*, intended for the comprehensive care of patients with advanced cancer, without possibility of cure, referred from other units of the Institute. Its institutional mission is “to promote and provide the highest quality palliative oncology care, with technical and humanitarian skill,” focusing on achieving the best possible quality of life for patients and their families through the control of physical, psychological, social, and spiritual symptoms.

The unit has a complete multidisciplinary structure, offering services in outpatient, home care, hospital inpatient, and emergency care settings. The inpatient area, where the data collection took place, consists of four floors, each with 14 beds distributed in double wards and single rooms, totaling 56 beds intended for symptom control, elective surgical procedures, and end-of-life care.

### Research Participants

The study population consisted of cancer patients hospitalized at *INCA*’s Cancer Hospital IV (HC IV), receiving palliative care, during the data collection period established in the research. Adult patients of both sexes, aged 18 years or older, diagnosed with advanced cancer and unresponsive to diseasemodifying treatment, as recorded in their medical records, were included. The inclusion criteria encompassed patients with preserved clinical and cognitive conditions to respond to the research instruments, with a minimum hospital stay of 24 hours, and who voluntarily agreed to participate by signing the Free Informed Consent Form (FICF).

Patients undergoing palliative sedation, with unstable clinical conditions, significant cognitive impairment, or neurological limitations that prevented them from understanding the questionnaire items were excluded.

For the sample size calculation, a confidence level of 90% and a sampling error of 5% were considered. Due to the characteristics of the population studied, an 80/20 ratio was adopted for a homogeneous population composed of cancer patients in palliative care. The number of hospitalizations in the unit in 2023 (n = 1,351) and the occupancy rate of 71% were used as a reference, resulting in a sample of 155 patients.

Data were obtained from the application of structured questionnaires and scales conducted at the bedside by the principal investigator and trained fellows, who followed a standardized protocol for approach and data collection. The average time for administering the instruments was approximately 25 minutes per participant, and the questionnaires were completed jointly by the researcher and the patient, ensuring better understanding of the items and minimizing response bias.

### Data Collection Instruments

Data collection was carried out through the application of a structured sociodemographic and clinical instrument, developed by the researcher, and two instruments validated for the Brazilian population: the GCQ and the ESAS.

The sociodemographic and clinical instrument included variables related to the participants’ characteristics, such as age, sex, religious beliefs, monthly income, type and stage of cancer, functionality, and the presence of devices and lesions. This information was obtained through a structured questionnaire and supplemented by reviewing medical records, ensuring the completeness and reliability of the data.

The GCQ was used to measure the level of patient comfort. The instrument consists of 48 items distributed across four dimensions: physical, psycho-spiritual, sociocultural, and environmental, evaluated on a four-point Likert scale (1 = strongly disagree to 4 = strongly agree). The total score is calculated by the arithmetic mean of the responses, ranging from 1 to 4, with higher values indicating greater comfort. The Brazilian version exhibits excellent internal consistency (Cronbach’s α = 0.89), consistent with previous studies conducted in hospital and palliative care populations^(17,18,19)^.

The ESAS was used to identify the intensity of the most common physical and emotional symptoms in patients with advanced cancer. The scale contains nine items that measure: pain, fatigue, nausea, depression, anxiety, drowsiness, appetite, well-being, and dyspnea, each evaluated on a numerical scale from 0 to 10, where 0 represents absence and 10 maximum symptom intensity^(20,21,22)^. The ESAS is widely used in clinical practice and research due to its ease of application, sensitivity to symptomatic variations, and cross-cultural validity, and is recommended by the European Association for Palliative Care and the American Society of Clinical Oncology^(22,23)^.

Both instruments were applied in the same session, in a calm environment, during the daytime, respecting the patient’s clinical and emotional conditions. The principal investigator oversaw all stages of data collection, ensuring uniformity in procedures and minimizing application biases.

## RESULTS

The sociodemographic characterization of the sample revealed a predominantly female profile, with an average age of 58 years, low monthly income, and most declared having some religious affiliation. Regarding clinical conditions, significant functional impairment and a high frequency of use of clinical devices were observed, findings consistent with the context of hospitalization in palliative care. Furthermore, diversity was observed regarding oncological diagnoses and conditions associated with disease progression (Table 1).

**Table 1:** Sociodemographic and clinical characterization of the participants (n = 155) – Rio de Janeiro, RJ, Brazil, 2025.

Variables	n (%)
**Sex**	
Female	106 (68.39)
Male	49 (31.61)
**Age group (years)**	
20 to 30	5 (3,23)
31 to 40	15 (9.68)
41 to 50	31 (20.00)
51 to 60	32 (20.65)
61 to 70	43 (28,29)
>70	26 (17.11)
**Has a religion**	
Yes	139 (89.68)
No	16 (10.32)
**Monthly income (minimum wage)**	
No income	27 (17.42)
Up to 1	86 (55.48)
1 to 2	29 (18.71)
3 to 4	11 (7.10)
>4	2 (1.29)
**Cancer diagnosis**	
Abdominal cancers	45 (29.03)
Gynecological	36 (23.23)
Breast	23 (14.84)
Others	51 (32.90)
**Functionality (KPS)**	
KPS 30%	93 (60.00)
KPS 40%	32 (20.65)
KPS 50%	25 (16,13)
KPS 60%	5 (3.23)
**Clinical devices/injuries**	
Stomas	37 (23.87)
Catheters/tubes	62 (40.00)
Vascular/subcutaneous access	149 (96.13)
Tumor wounds	29 (18.60)
Pressure ulcer	40 (25.81)

Legend: KPS = Karnofsky Performance Status.

The assessment of symptoms using the ESAS revealed a high symptomatic burden among hospitalized cancer patients receiving palliative care. Anxiety, loss of appetite, and depression presented the highest average intensity levels, followed by drowsiness and fatigue, as shown in Table 2.

**Table 2:** Characterization of the ESAS Scale (n = 155). – Río de Janeiro, RJ, Brasil, 2025.

Symptoms	Mean (±SD)	Median (IQR)	Minimum/Maximum
Pain	3.60 (3.55)	3 (10)	0/10
Tiredness	4.10 (3.28)	4 (10)	0/10
Nausea	2.48 (3.22)	0 (10)	0/10
Depression	4.63 (3.68)	5 (10)	0/10
Anxiety	5.51 (3.56)	7 (10)	0/10
Drowsiness	4.08 (3.53)	4 (10)	0/10
Loss of appetite	5.52 (3.42)	6 (10)	0/10
Dyspnea	1,35 (2,48)	0 (8)	0/9
Feeling of well-being	6.40 (3.18)	7 (10)	0/10
Total sum	37.68 (17.30)	38 (67)	1/76

Legend: SD = Standard deviation.

When correlating the symptoms assessed by the ESAS scale with the GCQ, a statistically significant negative correlation was observed between the scores of the ESAS scale and the GCQ for most of the symptoms assessed, with the exception of dyspnea. This finding shows that the increase in symptom intensity was associated with a reduction in the GCQ score (Table 3).

**Table 3:** Correlation between ESAS Scale symptoms and GCQ (n = 155) – Rio de Janeiro, RJ, Brazil, 2025.

Symptom Assessment Scale	General Comfort Questionnaire	
	*r*	*p*
Pain	–0.374	<0.001
Tiredness	–0.409	<0.001
Nausea	–0.247	0.002
Depression	–0.504	<0.001
Anxiety	–0.448	<0.001
Drowsiness	–0.172	0.031
Loss of appetite	–0.327	<0.001
Dyspnea	–0.105	0.193
Feeling of well-being	–0.535	<0.001
Total sum	–0.614	<0.001

Legend: *r* = Pearson’s correlation coefficient; *p* = statistical significance value.

No statistically significant associations were observed between comfort scores and sociodemographic variables. However, when analyzing comfort in the psycho-spiritual dimension, a statistically significant difference was observed between participants who declared having a religion and those who did not (p = 0.045). Individuals with some religious belief presented higher mean scores of psychospiritual comfort (mean: 51.28; SD: ± 7.65) compared to those without religion (mean: 47.06; SD ± 10.09), as shown in Table 4.

**Table 4:** Correlation between the psychospiritual domain of the GCQ and the religions of the study participants (n = 155) – Rio de Janeiro, RJ, Brazil, 2025.

Variables	Mean (±SD)	p-value
**Has a religion**		0.045^||^
Yes	51.28 (7,65)	
No	47.06 (10.09)	
**Religion**		0.090^‡^
Evangelical	51.10 (8.01)	
Catholic	52.0 (7.23)	
No religion	47.06 (10.09)	
Spiritist	51.63 (5.14)	
Others*	44.6 (10.62)	

Legend: ^||^Student’s t-test;

^‡^One-way ANOVA.

When assessing patient comfort by GCQ domain, it was observed that the Physical domain obtained the lowest mean scores (mean: 20.50; SD: ±6.24) and the Psychospiritual (mean: 50.84; SD: ±8.0) and Environmental (mean: 32.13; SD: ±5.30) domains obtained the highest means (Table 5).

**Table 5:** Characterization of comfort domains according to the GCQ (n = 155) – Río de Janeiro, RJ, Brasil, 2025.

Domains	Mean (±SD)	Median	P25	P75	Minimum/Maximum
Physical	20.50 (6.24)	20	10	34	10/36
Environmental	32.13 (5.30)	32	22	42	15/43
Sociocultural	22.10 (4.60)	21	15	33	9/36
Psychospiritual	50.84 (8.0)	51	30	67	25/69
Overall comfort	125.58 (18.52)	126	89	167	64/174

Legend: SD = Standard deviation.

## DISCUSSION

The results of this research consistently reveal that increased intensity of physical symptoms is inversely associated with the overall and physical comfort perceived by hospitalized cancer patients in palliative care. This negative correlation, observed for symptoms such as pain and fatigue, confirms the hypothesis that discomfort is a direct consequence of symptomatic accumulation, impacting not only the physical well-being, but also the patient’s emotional and spiritual well-being. Based on this observation, the premise of Kolcaba’s Theory of Comfort is reaffirmed, according to which comfort results from the satisfaction of human needs in multiple dimensions, with the physical dimension being the most sensitive to changes resulting from advanced disease^(16,17)^.

The finding that pain and fatigue showed negative correlations with physical comfort is consistent with studies that identify these symptoms as the most disabling and prevalent in palliative care patients^(18,19,20)^. In an international analysis involving more than 1,000 patients, the authors demonstrated that inadequate pain management compromises adherence to therapies and reduces the perception of well-being^(19)^. Similarly, they observed that effective symptom control was one of the main predictors of satisfaction with the care received, indicating that interventions targeting pain and fatigue have a direct impact on the perception of comfort^(20)^. Regarding dyspnea, although not statistically significant in this study, it is a clinically relevant symptom, especially because it is a distressing symptom associated with a perception of vulnerability and fear of death^(21)^.

Qualitative studies indicate that the sensation of shortness of breath tends to exacerbate psychological distress and increase the feeling of loss of control. This subjective dimension reinforces the need for integrated approaches that combine pharmacological therapies with emotional and spiritual support, broadening the scope of care beyond the physiological control of symptoms^(18,19,20 21,22)^. The relationship between comfort and emotional symptoms, such as anxiety and depression, also emerged significantly in the results. The observed negative correlation indicates that emotional distress acts as a mediator of overall discomfort, interfering with how the patient experiences physical symptoms and the hospitalization itself. Recent research with the ESAS emphasize that psychological distress strongly influences the total score on the scale, amplifying the perception of fatigue and pain^(19)^. Psychological and empathetic communication interventions promote not only emotional improvement, but also increased physical and social comfort^(22)^.

Another relevant aspect is that the average overall comfort score observed in this study was higher than that reported in international studies with similar samples, which suggests the effectiveness of the care model adopted by Hospital do Câncer IV/INCA. This unit follows the principle of patient- and familycentered care, with strong multidisciplinary integration, which aligns with the recommendations of *World Health Organization* and from *American Society of Clinical Oncology* ^(23)^. The constant presence of nursing, medical, nutrition, psychology, physiotherapy, and chaplaincy teams promotes comprehensive care, fostering both symptom relief and the patient’s emotional and spiritual strengthening.

The study observed that the physical domain presented the lowest average among the domains evaluated, indicating greater impairment and reflecting the negative impact of the multiple physical symptoms experienced by patients in palliative care.

The psycho-spiritual dimension, although not the primary focus of the correlation, stood out among the highest scores of the GCQ. This result corroborates evidence that spirituality acts as a positive mediator of comfort, allowing one to reframe pain and accept finitude^(21,22)^. In a recent review^(16)^, the authors demonstrated that interventions based on Kolcaba’s Comfort Theory are effective in reducing anxiety and increasing inner peace, especially in end-of-life contexts. These findings reiterate that comfort transcends physical relief, being a complex phenomenon that integrates existential, social, and emotional dimensions.

From a methodological point of view, this study advances the empirical understanding of Comfort Theory by statistically confirming the interdependence between discomfort and symptomatic burden. The combination of instruments ESAS and GCQ proved to be sensitive and complementary in assessing the clinical and subjective state of patients, allowing for the accurate identification of areas of greatest vulnerability. Recent studies reinforce the value of standardized instruments for monitoring comfort and overall distress, recommending their routine use in healthcare practice^(20,21,22)^.

Among the study’s limitations, the following stand out: the cross-sectional design, which hinders inferences of causality, and the use of a non-probabilistic sample, restricted to a single institution. Despite this, the homogeneity of the sample and the standardization of the collection process lend internal robustness to the results. As a step forward for nursing, the findings reaffirm the importance of incorporating comfort measurement as a quality care indicator, as well as supporting the development of evidencebased care protocols. The integration of symptom assessment and comfort can guide clinical decisions, promote relief from suffering, and strengthen the humanization of palliative care^(19,20,21,22,23)^.

The results of this study have direct and significant implications for nursing practice in oncological palliative care, showing that the intensity of physical and emotional symptoms is strongly associated with a decrease in perceived comfort among patients. This finding reinforces the need for nurses to adopt comfort as a clinical indicator of quality of care, integrating its assessment into the nursing process, especially in the diagnostic and care planning stages.

The combined use of ESAS and GCQ constitutes a practical, fast, and validated strategy for the continuous monitoring of patient well-being. The systematic application of these instruments can guide the prioritization of nursing interventions, promote timely symptom management, and support evidencebased clinical decision-making. From an ethical and humanistic standpoint, the findings reaffirm that nursing professionals should recognize comfort as an essential human right, especially in end-of-life situations. A sensitive, empathetic, and personcentered approach enhances the effectiveness of palliative care and strengthens the therapeutic bond, promoting the patient’s dignity and autonomy until the end of life.

Furthermore, the study contributes to the improvement of continuing health education practices by demonstrating that comfort measurement can be incorporated as an essential competency in care protocols and routine healthcare practices. Ongoing training for nursing teams, focusing on the integrated assessment of symptoms and comfort, enhances the quality and safety of care provided in public and private healthcare institutions.

## CONCLUSION

The study showed that increased intensity of physical symptoms significantly reduces the overall comfort of hospitalized cancer patients in palliative care. These findings confirm that the effective management of physical symptoms is a central element in maintaining well-being and dignity in the dying process, and reinforce that comfort is not only a desirable outcome of care, but also a sensitive clinical indicator of the quality of care provided.

The results reinforce the relevance of the nursing team, within an interdisciplinary context, in conducting therapeutic strategies that combine technical competence, ethical sensitivity, and humanized care. The systematic incorporation of comfort and symptom assessment into care routines allows for the early identification of complications and the planning of individualized interventions, with continuous monitoring of results.

This way, the research contributes to consolidating comfort as a measurable parameter of the quality of nursing care provided to cancer patients in palliative care.

## Data Availability

All the data supporting the results of this study were published in the article itself.
