# Supplementary material for: Correlation between physical symptoms and overall comfort in hospitalized cancer patients in palliative care
Source: Rev Esc Enferm USP. 2026 Jul 6;60:e20250511. doi: 10.1590/1980-220X-REEUSP-2025-0511en (PMC13337296; doi:10.1590/1980-220X-REEUSP-2025-0511en)
Supplement: Translation

# Translation: Correlação entre sintomas físicos e conforto geral em pacientes com câncer hospitalizados em cuidados paliativos*

**DOI:** 10.1590/1980-220X-REEUSP-2025-0511pt

**Authors:** Ana Luísa Teixeira da Costa Durante, Lívia Costa de Oliveira, Andrea dos Santos Garcia, Sônia Regina de Souza, Daniel Aragão Machado, Carlos Roberto Lyra da Silva

**Affiliations:** 1 Instituto Nacional do Câncer, Rio de Janeiro, RJ, Brasil.; 2 Universidade Federal do Rio de Janeiro, Escola de Enfermagem Anna Nery, Departamento de Enfermagem Médico-Cirúrgico, Rio de Janeiro, RJ, Brasil.; 3 Universidade Federal do Estado do Rio de Janeiro, Escola de Enfermagem Alfredo Pinto, Programa de Pós-Graduação em Enfermagem e Biociências, Rio de Janeiro, RJ, Brasil.; 4 Universidade Federal do Estado do Rio de Janeiro, Programa de Pós-Graduação em Saúde e Tecnologia no Espaço Hospitalar, Rio de Janeiro, RJ, Brasil.

**Keywords:** Cuidados paliativos, Conforto do paciente, Enfermagem oncológica, Avaliação de sintomas, Qualidade de vida

## Abstract

**Objetivo:**

Analisar a correlação entre a intensidade dos sintomas físicos e os níveis de conforto geral em pacientes com câncer hospitalizados em cuidados paliativos.

**Método:**

Estudo quantitativo, descritivo e analítico, realizado com 155 pacientes internados em unidades de cuidados paliativos de um hospital universitário. Utilizaram-se o Questionário de Conforto Geral (QCG) e a Escala de Avaliação de Sintomas de Edmonton (ESAS). Os dados foram analisados por estatística descritiva e inferencial, utilizando correlação de Pearson (p ≤ 0,05).

**Resultados:**

A avaliação dos sintomas pela ESAS evidenciou elevada carga sintomática, destacando-se ansiedade, falta de apetite e depressão como os sintomas de maior intensidade média. Verificou-se correlação negativa estatisticamente significativa entre a intensidade da maioria dos sintomas e o conforto geral, indicando que o aumento da carga sintomática esteve associado à redução do conforto.

**Conclusão:**

O aumento da intensidade dos sintomas físicos está associado à redução significativa do conforto geral, reforçando a importância de estratégias eficazes para o manejo desses sintomas na prática da enfermagem paliativa.

## INTRODUÇÃO

O conforto é um dos pilares da prática de enfermagem em cuidados paliativos e representa um indicador essencial da qualidade assistencial. A Teoria do Conforto compreende o conforto como um estado dinâmico de alívio, tranquilidade e transcendência nas dimensões física, psicoespiritual, sociocultural e ambiental. Entre essas, a dimensão física é a mais diretamente afetada pela presença de sintomas, os quais comprometem o bem-estar e a percepção de qualidade de vida^(1)^. Pacientes com câncer hospitalizados em fase paliativa vivenciam múltiplos sintomas físicos, tais como dor, fadiga, dispneia e inapetência, que interferem diretamente no conforto e na experiência de cuidado^(2)^.

O avanço da doença oncológica geralmente intensifica a presença de sintomas múltiplos e de difícil controle, ocasionando sofrimento físico, emocional e existencial. O manejo desses sintomas constitui uma prioridade nos cuidados paliativos, pois está diretamente relacionado à promoção do conforto e à qualidade de vida^(2)^. Nesse contexto, a mensuração sistemática da intensidade dos sintomas torna-se essencial para orientar intervenções individualizadas e avaliar a efetividade das práticas de enfermagem voltadas ao alívio e bem-estar^(3)^.

O conforto físico representa a experiência de alívio e tranquilidade decorrente da satisfação de necessidades corporais básicas e do controle da dor e de outros sintomas^(1,2,3)^. A ausência de conforto traduz-se em desconforto, fadiga e sofrimento, interferindo no equilíbrio homeostático e emocional do paciente com câncer^(4)^. Assim, a avaliação do conforto deve ser integrada à rotina assistencial, permitindo identificar precocemente as alterações que impactam negativamente o estado geral e a resposta terapêutica ao tratamento^(5)^.

Estudos recentes com pacientes em cuidados paliativos apontam correlação significativa entre sintomas físicos intensos e baixos níveis de conforto geral, sugerindo que o desconforto é uma consequência direta do acúmulo sintomático^(3)^. A literatura evidencia que quanto maior a intensidade da dor e da fadiga, menores são os escores de conforto físico e global^(4,5,6)^. Esse achado reforça o papel do enfermeiro na monitorização contínua dos sintomas, na aplicação de escalas padronizadas como a *Edmonton Symptom Assessment Scale (ESAS)* e na adoção de condutas baseadas em evidências para o controle sintomático^(7,8,9)^.

A utilização do *Questionário de Conforto Geral (QCG)* no contexto dos cuidados paliativos permite compreender o conforto como fenômeno multidimensional e avaliar de forma integrada suas dimensões física, psicoespiritual, sociocultural e ambiental. Em pacientes com câncer hospitalizados, o QCG tem demonstrado sensibilidade para detectar variações no bemestar diante das intervenções de enfermagem, possibilitando identificar os fatores que mais comprometem o conforto^(3,4,5)^. Essa ferramenta auxilia na formulação de planos de cuidado centrados nas necessidades do paciente, especialmente no controle de sintomas que afetam o corpo e o espírito simultaneamente^(3,4,5,6,7)^.

Dessa forma, investigar a relação entre sintomas físicos e conforto geral contribui não apenas para o aprimoramento da prática assistencial, mas também para o fortalecimento de indicadores de qualidade em cuidados paliativos. Ao evidenciar que o desconforto físico está fortemente associado à intensidade sintomática, torna-se possível direcionar esforços interdisciplinares para intervenções precoces e eficazes^(8,9,10,11,12)^. A integração entre avaliação de sintomas e mensuração do conforto deve ser considerada um componente essencial das boas práticas em enfermagem paliativa, com impacto direto na dignidade, no bem-estar e na humanização do cuidado^(13)^.

Embora a relação entre sintomas físicos e o conforto em pacientes com câncer já seja reconhecida na literatura, observase escassez de estudos que investiguem essas associações de forma correlacional em indivíduos hospitalizados em cuidados paliativos, especialmente no contexto brasileiro e utilizando instrumentos validados de maneira combinada. A maioria das investigações concentra-se em contextos ambulatoriais ou em fases específicas da doença^(7,9,14)^. Assim, o presente estudo teve como objetivo analisar as correlações entre a intensidade dos sintomas físicos e os níveis de conforto geral em pacientes com câncer hospitalizados em cuidados paliativos.

## MÉTODO

### Tipo do Estudo

Trata-se de um estudo quantitativo, observacional, descritivo e analítico, com delineamento transversal, fundamentado na Teoria do Conforto de Kolcaba^(1)^.

O desenho transversal possibilitou a coleta de dados em um único momento da internação, favorecendo a análise das inter-relações entre os sintomas avaliados pela *ESAS* ^(3,15,16,17,18)^ e os níveis de conforto mensurados pelo QCG^(1,11,12)^.

As análises da associação entre conforto geral e variáveis sociodemográficas foram realizadas por meio de testes de comparação de médias (teste t de Student ou análise de variância – ANOVA, conforme o tipo de variável), enquanto as relações entre a intensidade dos sintomas e os níveis de conforto geral foram examinadas por meio do coeficiente de correlação de Pearson.

### Local da Pesquisa

O estudo foi desenvolvido no Hospital do Câncer IV (HC IV) do Instituto Nacional de Câncer (INCA), localizado na cidade do Rio de Janeiro, Brasil. O INCA é uma instituição vinculada ao Ministério da Saúde e constitui-se como o principal centro público de referência nacional em prevenção e controle do câncer, com quatro unidades assistenciais distribuídas na cidade: HC I, HC II, HC III e HC IV.

O HC IV é a unidade de cuidados paliativos do INCA, destinada ao atendimento integral de pacientes com câncer avançado, sem possibilidade de cura, encaminhados das demais unidades do Instituto. Sua missão institucional é “promover e prover cuidados paliativos oncológicos da mais alta qualidade, com habilidade técnica e humanitária”, tendo como foco a obtenção da melhor qualidade de vida possível para pacientes e familiares por meio do controle de sintomas físicos, psicológicos, sociais e espirituais.

A unidade dispõe de estrutura multiprofissional completa, com atendimentos nas modalidades ambulatorial, domiciliar, internação hospitalar e pronto atendimento. A área de internação, onde se desenvolveu a coleta de dados, é composta por quatro andares, cada um com 14 leitos distribuídos em enfermarias duplas e quartos individuais, totalizando 56 leitos destinados ao controle de sintomas, realização de procedimentos cirúrgicos eletivos e cuidados no fim de vida.

### Participantes da Pesquisa

A população do estudo foi composta por pacientes com câncer hospitalizados no Hospital do Câncer IV (HC IV) do Instituto Nacional de Câncer (INCA), em cuidados paliativos, durante o período de coleta estabelecido na pesquisa. Foram incluídos pacientes adultos, de ambos os sexos, com idade igual ou superior a 18 anos, diagnosticados com câncer avançado e em fase não responsiva ao tratamento modificador da doença, conforme registro no prontuário clínico. Os critérios de inclusão abrangeram pacientes com condições clínicas e cognitivas preservadas para responder aos instrumentos de pesquisa, com tempo mínimo de internação de 24 horas e que aceitaram participar voluntariamente mediante assinatura do Termo de Consentimento Livre e Esclarecido (TCLE).

Foram excluídos os pacientes em sedação paliativa, com quadro clínico instável, deterioração cognitiva significativa ou que apresentassem limitações neurológicas que impedissem a compreensão dos itens dos questionários.

Para o cálculo amostral, considerou-se um nível de confiança de 90% e erro amostral de 5%. Em razão das características da população investigada, adotou-se a proporção de 80/20 para população homogênea, composta por pacientes com câncer em cuidados paliativos. Utilizou-se como referência o número de internações na unidade no ano de 2023 (n = 1.351) e a taxa de ocupação de 71%, resultando em uma amostra de 155 pacientes.

Os dados foram obtidos a partir de aplicação de questionários estruturados e escalas realizadas à beira-leito pela pesquisadora responsável e por bolsistas treinados, que seguiram um protocolo padronizado de abordagem e coleta. O tempo médio para aplicação dos instrumentos foi de aproximadamente 25 minutos por participante, e os questionários foram preenchidos de forma conjunta entre pesquisador e paciente, garantindo melhor compreensão dos itens e minimização de vieses de resposta.

### Instrumentos de Coleta de Dados

A coleta de dados foi realizada por meio da aplicação de um instrumento sociodemográfico e clínico estruturado, elaborado pela pesquisadora, e de dois instrumentos validados para a população brasileira: o QCG e a *ESAS*.

O instrumento sociodemográfico e clínico contemplou variáveis relacionadas à caracterização dos participantes, como idade, sexo, crença religiosa, renda mensal, tipo e estágio do câncer, funcionalidade e presença de dispositivos e lesões. Essas informações foram obtidas por aplicação de questionário estruturado e complementadas pela consulta aos prontuários clínicos, garantindo a completude e a fidedignidade dos dados.

O QCG foi utilizado para mensurar o nível de conforto dos pacientes. O instrumento é composto por 48 itens distribuídos em quatro dimensões: física, psicoespiritual, sociocultural e ambiental, avaliados em uma escala Likert de quatro pontos (1 = discordo totalmente a 4 = concordo totalmente). O escore total é calculado pela média aritmética das respostas, variando de 1 a 4, sendo valores mais altos indicativos de maior conforto. A versão brasileira apresenta excelente consistência interna (α de Cronbach = 0,89), conforme estudos prévios realizados em populações hospitalares e paliativas^(17,18,19)^.

A *ESAS* foi empregada para identificar a intensidade dos sintomas físicos e emocionais mais comuns em pacientes com câncer avançado. A escala contém nove itens, que mensuram: dor, fadiga, náusea, depressão, ansiedade, sonolência, apetite, bem-estar e dispneia, cada um avaliado em uma escala numérica de 0 a 10, em que 0 representa ausência e 10 máxima intensidade do sintoma^(20,21,22)^. A *ESAS* é amplamente utilizada na prática clínica e em pesquisas por sua facilidade de aplicação, sensibilidade para variações sintomáticas e validade transcultural, sendo recomendada pela *European Association for Palliative Care* e pela *American Society of Clinical Oncology* ^(22,23)^.

Ambos os instrumentos foram aplicados no mesmo encontro, em ambiente tranquilo, durante o período diurno, respeitando as condições clínicas e emocionais do paciente. A pesquisadora principal supervisionou todas as etapas da coleta, garantindo uniformidade nos procedimentos e minimização de vieses de aplicação.

## RESULTADOS

A caracterização sociodemográfica da amostra revelou um perfil predominantemente feminino, idade média de 58 anos, baixa renda mensal e a maioria declarou possuir algum vínculo religioso. Com relação às condições clínicas, observaram-se acentuado comprometimento funcional e elevada frequência de utilização de dispositivos clínicos, achados compatíveis com o contexto de internação em cuidados paliativos. Constatou-se ainda diversidade quanto aos diagnósticos oncológicos e às condições associadas à progressão da doença (Tabela 1).

**Tabela 1:** Caracterização sociodemográfica e clínica dos participantes (n = 155) – Rio de Janeiro, RJ, Brasil, 2025.

Variáveis	n (%)
**Sexo**	
Feminino	106 (68,39)
Masculino	49 (31,61)
**Faixa etária (anos)**	
20 a 30	5 (3,23)
31 a 40	15 (9,68)
41 a 50	31 (20,00)
51 a 60	32 (20,65)
61 a 70	43 (28,29)
>70	26 (17,11)
**Possui religião**	
Sim	139 (89,68)
Não	16 (10,32)
**Renda mensal (salário – mínimo)**	
Nenhuma renda	27 (17,42)
Até 1	86 (55,48)
1 a 2	29 (18,71)
3 a 4	11 (7,10)
>4	2 (1,29)
**Diagnóstico oncológico**	
Cânceres abdominais	45 (29,03)
Ginecológicos	36 (23,23)
Mama	23 (14,84)
Outros	51 (32,90)
**Funcionalidade (KPS)**	
KPS 30%	93 (60,00)
KPS 40%	32 (20,65)
KPS 50%	25 (16,13)
KPS 60%	5 (3,23)
**Dispositivos clínicos/lesões**	
Estomas	37 (23,87)
Cateteres/drenos	62 (40,00)
Acesso vascular/subcutâneo	149 (96,13)
Feridas tumorais	29 (18,60)
Lesão por pressão	40 (25,81)

Legenda: KPS = Karnofsky Performance Status.

A avaliação dos sintomas por meio da aplicação da ESAS evidenciou elevada carga sintomática entre os pacientes com câncer hospitalizados em cuidados paliativos. Ansiedade, falta de apetite e depressão apresentaram as maiores médias de intensidade, seguidas por sonolência e fadiga, conforme apresentado na Tabela 2.

**Tabela 2:** Caracterização da Escala ESAS (n = 155). – Rio de Janeiro, RJ, Brasil, 2025.

Sintomas	Média (±DP)	Mediana (IIQ)	Mínimo/Máximo
Dor	3,60 (3,55)	3 (10)	0/10
Cansaço	4,10 (3,28)	4 (10)	0/10
Náusea	2,48 (3,22)	0 (10)	0/10
Depressão	4,63 (3,68)	5 (10)	0/10
Ansiedade	5,51 (3,56)	7 (10)	0/10
Sonolência	4,08 (3,53)	4 (10)	0/10
Falta de apetite	5,52 (3,42)	6 (10)	0/10
Dispneia	1,35 (2,48)	0 (8)	0/9
Sensação de bem-estar	6,40 (3,18)	7 (10)	0/10
Somatório total	37,68 (17,30)	38 (67)	1/76

Legenda: DP = desvio-padrão.

Ao correlacionar os sintomas avaliados pela escala ESAS com o QCG foi possível observar correlação negativa estatisticamente significativa entre os escores da escala ESAS e do QCG para a maioria dos sintomas avaliados, com exceção da dispneia. Esse achado evidencia que o aumento da intensidade dos sintomas esteve associado à redução da pontuação no QCG (Tabela 3).

**Tabela 3:** Correlação entre os sintomas da Escala ESAS e o QCG (n = 155) – Rio de Janeiro, RJ, Brasil, 2025.

Escala de Avaliação de Sintomas	Questionário de Conforto Geral	
	*r*	*p*
Dor	–0,374	<0,001
Cansaço	–0,409	<0,001
Náusea	–0,247	0,002
Depressão	–0,504	<0,001
Ansiedade	–0,448	<0,001
Sonolência	–0,172	0,031
Falta de apetite	–0,327	<0,001
Dispneia	–0,105	0,193
Sensação de bem-estar	–0,535	<0,001
Somatório total	–0,614	<0,001

Legenda: *r* = coeficiente de correlação de Pearson; *p* = valor de significância estatística.

Não foram observadas associações estatisticamente significativas entre os escores de conforto e as variáveis sociodemográficas. Entretanto, ao analisar o conforto na dimensão psicoespiritual observou-se diferença estatisticamente significativa entre participantes que declararam possuir religião e os que não possuíam (p = 0,045). Indivíduos com alguma crença religiosa apresentaram escores médios mais elevados de conforto psicoespiritual (média: 51,28; DP: ± 7,65) em comparação àqueles sem religião (média: 47,06; DP ± 10,09), conforme apresentado na Tabela 4.

**Tabela 4:** Correlação do domínio psicoespiritual do QCG e religiões dos participantes do estudo (n = 155) – Rio de Janeiro, RJ, Brasil, 2025.

Variáveis	Média (±DP)	Valor de p
**Possui religião**		0,045^||^
Sim	51,28 (7,65)	
Não	47,06 (10,09)	
**Religião**		0,090^‡^
Evangélica	51,10 (8,01)	
Católica	52,0 (7,23)	
Sem religião	47,06 (10,09)	
Espírita	51,63 (5,14)	
Outras*	44,6 (10,62)	

Legenda: ^||^Teste t de Student;

^‡^ANOVA a um fator.

Ao avaliar o conforto dos pacientes por domínio do QCG, observou-se que o domínio Físico obteve os menores escores médios (média: 20,50; DP: ±6,24) e os domínios Psicoespiritual (média: 50,84; DP: ±8,0) e Ambiental (média: 32,13; DP: ±5,30) obtiveram as maiores médias (Tabela 5).

**Tabela 5:** Caracterização dos domínios do conforto segundo o QCG (n = 155) – Rio de Janeiro, RJ, Brasil, 2025.

Domínios	Média (±DP)	Mediana	P25	P75	Mínimo/Máximo
Físico	20,50 (6,24)	20	10	34	10/36
Ambiental	32,13 (5,30)	32	22	42	15/43
Sociocultural	22,10 (4,60)	21	15	33	9/36
Psicoespiritual	50,84 (8,0)	51	30	67	25/69
Conforto geral	125,58 (18,52)	126	89	167	64/174

Legenda: DP = desvio-padrão.

## DISCUSSÃO

Os resultados desta pesquisa revelam de forma consistente que o aumento da intensidade dos sintomas físicos está inversamente associado ao conforto geral e físico percebido por pacientes com câncer hospitalizados em cuidados paliativos. Essa correlação negativa, observada para sintomas como dor e fadiga, confirma a hipótese de que o desconforto é uma consequência direta do acúmulo sintomático, impactando não apenas o bem-estar físico, mas também o emocional e espiritual do paciente. A partir dessa constatação, reafirma-se o pressuposto da Teoria do Conforto de Kolcaba, segundo a qual o conforto resulta da satisfação de necessidades humanas em múltiplas dimensões, sendo a física a mais sensível às alterações decorrentes da doença avançada^(16,17)^.

O achado de que dor e fadiga apresentaram correlações negativas com o conforto físico está em consonância com estudos que identificam esses sintomas como os mais incapacitantes e prevalentes em pacientes em cuidados paliativos^(18,19,20)^. Em uma análise internacional envolvendo mais de 1.000 pacientes, os autores demonstraram que o manejo inadequado da dor compromete a adesão às terapias e reduz a percepção de bemestar^(19)^. Da mesma forma, observaram que o controle sintomático efetivo foi um dos principais preditores de satisfação com o cuidado recebido, indicando que intervenções direcionadas à dor e à fadiga têm impacto direto na percepção de conforto^(20)^. Em relação à dispneia, embora sem significância estatística neste estudo, trata-se de um sintoma clinicamente relevante, especialmente por ser um sintoma angustiante associado à percepção de vulnerabilidade e medo de morrer^(21)^.

Estudos qualitativos apontam que a sensação de falta de ar tende a exacerbar o sofrimento psicológico e aumentar o sentimento de perda de controle. Essa dimensão subjetiva reforça a necessidade de abordagens integradas que associem terapias farmacológicas ao suporte emocional e espiritual, ampliando o escopo do cuidado para além do controle fisiológico do sintoma^(18,19,20,21,22)^. A relação entre conforto e sintomas emocionais, como ansiedade e depressão, também emergiu de forma expressiva nos resultados. A correlação negativa observada indica que o sofrimento emocional atua como mediador do desconforto global, interferindo na forma como o paciente vivencia os sintomas físicos e a própria hospitalização. Pesquisas recentes com a *ESAS* reforçam que o sofrimento psicológico influencia fortemente o escore total da escala, ampliando a percepção de fadiga e dor^(19)^. Intervenções psicológicas e de comunicação empática promovem não apenas melhora emocional, mas também incremento no conforto físico e social^(22)^.

Outro aspecto relevante é que o escore médio de conforto geral observado neste estudo foi superior ao relatado em estudos internacionais com amostras semelhantes, o que sugere a eficácia do modelo assistencial adotado pelo Hospital do Câncer IV/INCA. Essa unidade segue o princípio do cuidado centrado no paciente e na família, com forte integração multiprofissional, o que se alinha às recomendações da *World Health Organization* e da *American Society of Clinical Oncology* ^(23)^. A presença constante de equipes de enfermagem, médica, nutrição, psicologia, fisioterapia e capelania favorece uma assistência integral, promovendo tanto o alívio de sintomas quanto o fortalecimento emocional e espiritual do paciente.

Observou-se no estudo que o domínio físico apresentou a menor média entre os domínios avaliados, indicando maior comprometimento e refletindo o impacto negativo dos múltiplos sintomas físicos experimentados pelos pacientes em cuidados paliativos.

A dimensão psicoespiritual, embora não tenha sido o foco principal da correlação, destacou-se entre os escores mais elevados do *QCG*. Esse resultado corrobora evidências de que a espiritualidade atua como mediadora positiva do conforto, permitindo ressignificar a dor e aceitar a finitude^(21,22)^. Em revisão recente^(16)^, os autores demonstraram que intervenções baseadas na Teoria do Conforto de Kolcaba são eficazes para reduzir ansiedade e aumentar o senso de paz interior, especialmente em contextos de fim de vida. Tais achados reiteram que o conforto transcende o alívio físico, sendo um fenômeno complexo que integra dimensões existenciais, sociais e emocionais.

Do ponto de vista metodológico, este estudo avança na compreensão empírica da Teoria do Conforto, ao confirmar estatisticamente a interdependência entre desconforto e carga sintomática. A combinação dos instrumentos *ESAS* e *QCG* mostrou-se sensível e complementar para avaliar o estado clínico e subjetivo dos pacientes, permitindo identificar de forma acurada as áreas de maior vulnerabilidade. Estudos recentes reforçam o valor de instrumentos padronizados para o monitoramento do conforto e do sofrimento total, recomendando seu uso rotineiro na prática assistencial^(20,21,22)^.

Entre as limitações do estudo, destacam-se o delineamento transversal, que impossibilita inferências de causalidade, e o uso de uma amostra não probabilística, restrita a uma única instituição. Apesar disso, a homogeneidade da amostra e a padronização da coleta conferem robustez interna aos resultados. Como avanço para a enfermagem, os achados reafirmam a importância de incorporar a mensuração do conforto como indicador assistencial de qualidade, além de subsidiar a construção de protocolos de cuidados baseados em evidências. A integração entre avaliação de sintomas e conforto pode orientar decisões clínicas, promover alívio do sofrimento e fortalecer a humanização do cuidado paliativo^(19,20,21,22,23)^.

Os resultados deste estudo apresentam implicações diretas e significativas para a prática de enfermagem em cuidados paliativos oncológicos, ao evidenciarem que a intensidade dos sintomas físicos e emocionais está fortemente associada à diminuição do conforto percebido pelos pacientes. Essa constatação reforça a necessidade de que os enfermeiros adotem o conforto como indicador clínico de qualidade assistencial, integrando sua avaliação ao processo de enfermagem, em especial nas etapas de diagnóstico e planejamento do cuidado.

A utilização conjunta da ESAS e do QCG constitui uma estratégia prática, rápida e validada para o monitoramento contínuo do bem-estar do paciente. A aplicação sistemática desses instrumentos pode orientar a priorização de intervenções de enfermagem, promover o manejo oportuno dos sintomas e apoiar a tomada de decisão clínica baseada em evidências. Do ponto de vista ético e humanístico, os achados reafirmam que os profissionais de enfermagem devem reconhecer o conforto como direito humano essencial, sobretudo em situações de terminalidade. A abordagem sensível, empática e centrada na pessoa amplia a efetividade das ações paliativas e fortalece o vínculo terapêutico, favorecendo a dignidade e a autonomia do paciente até o final da vida.

Além disso, o estudo contribui para o aperfeiçoamento das práticas de educação permanente em saúde, ao demonstrar que a mensuração do conforto pode ser incorporada como competência essencial nos protocolos de cuidado e nas rotinas assistenciais. A formação continuada das equipes de enfermagem, com foco na avaliação integrada de sintomas e conforto, potencializa a qualidade e a segurança do cuidado prestado em instituições de saúde públicas e privadas.

## CONCLUSÃO

O estudo evidenciou que o aumento da intensidade dos sintomas físicos reduz significativamente o conforto geral de pacientes com câncer hospitalizados em cuidados paliativos. Esses achados confirmam que o manejo efetivo dos sintomas físicos constitui elemento central para a manutenção do bem-estar e da dignidade no processo de morrer, além de reforçar que o conforto não é apenas um resultado desejável do cuidado, mas um indicador clínico sensível à qualidade da assistência prestada.

Os resultados reforçam a relevância da equipe de enfermagem, no contexto interdisciplinar, na condução de estratégias terapêuticas que aliam competência técnica, sensibilidade ética e cuidado humanizado. A incorporação sistemática da avaliação de conforto e de sintomas nas rotinas assistenciais permite a identificação precoce de agravos e o planejamento de intervenções individualizadas, com monitoramento contínuo dos resultados.

Dessa forma, a pesquisa contribui para consolidar o conforto como parâmetro mensurável da qualidade do cuidado de enfermagem prestado a pacientes com câncer em cuidados paliativos.

## Data Availability

Todo o conjunto de dados que dá suporte aos resultados deste estudo foi publicado no próprio artigo.

# Translation: Correlación entre los síntomas físicos y el bienestar general en pacientes oncológicos hospitalizados en cuidados paliativos*

**DOI:** 10.1590/1980-220X-REEUSP-2025-0511es

**Authors:** Ana Luísa Teixeira da Costa Durante, Lívia Costa de Oliveira, Andrea dos Santos Garcia, Sônia Regina de Souza, Daniel Aragão Machado, Carlos Roberto Lyra da Silva

**Affiliations:** 1 Instituto Nacional do Câncer, Rio de Janeiro, RJ, Brasil.; 2 Universidade Federal do Rio de Janeiro, Escola de Enfermagem Anna Nery, Departamento de Enfermagem Médico-Cirúrgico, Rio de Janeiro, RJ, Brasil.; 3 Universidade Federal do Estado do Rio de Janeiro, Escola de Enfermagem Alfredo Pinto, Programa de Pós-Graduação em Enfermagem e Biociências, Rio de Janeiro, RJ, Brasil.; 4 Universidade Federal do Estado do Rio de Janeiro, Programa de Pós-Graduação em Saúde e Tecnologia no Espaço Hospitalar, Rio de Janeiro, RJ, Brasil.

**Keywords:** Cuidados paliativos, Comodidad del paciente, Enfermería Oncológica, Evaluación de síntomas, Calidad de Vida

## Abstract

**Objetivo:**

Analizar la correlación entre la intensidad de los síntomas físicos y el nivel general de bienestar en pacientes oncológicos hospitalizados que reciben cuidados paliativos.

**Método:**

Este estudio cuantitativo, descriptivo y analítico se realizó con 155 pacientes ingresados en unidades de cuidados paliativos de un hospital universitario. Se utilizaron el Cuestionario de Confort General (CCG) y la Escala de Evaluación de Síntomas de Edmonton (ESAS). Los datos se analizaron utilizando estadística descriptiva e inferencial, empleando la correlación de Pearson (p ≤ 0,05).

**Resultados:**

La evaluación de síntomas de la escala ESAS reveló una elevada carga sintomática, destacando la ansiedad, la pérdida de apetito y la depresión como los síntomas con la mayor intensidad media. Se encontró una correlación negativa estadísticamente significativa entre la intensidad de la mayoría de los síntomas y el bienestar general, lo que indica que una mayor carga de síntomas se asoció con una menor comodidad.

**Conclusión:**

El aumento de la intensidad de los síntomas físicos se asocia con una reducción significativa del bienestar general, lo que refuerza la importancia de contar con estrategias eficaces para el manejo de estos síntomas en la práctica de enfermería paliativa.

## INTRODUCCIÓN

El bienestar es uno de los pilares de la práctica de enfermería en cuidados paliativos y representa un indicador esencial de la calidad asistencial. La Teoría del Bienestar entiende el bienestar como un estado dinámico de alivio, tranquilidad y trascendencia en las dimensiones física, psicoespiritual, sociocultural y ambiental. Entre estas, la dimensión física es la más directamente afectada por la presencia de síntomas, los cuales comprometen el bienestar y la percepción de la calidad de vida^(1)^. Los pacientes con cáncer hospitalizados en fase paliativa experimentan múltiples síntomas físicos, tales como dolor, fatiga, disnea y falta de apetito, que interfieren directamente en el confort y en la experiencia de la atención^(2)^.

El avance de la enfermedad oncológica suele intensificar la presencia de múltiples síntomas difíciles de controlar, lo que provoca sufrimiento físico, emocional y existencial. El manejo de estos síntomas constituye una prioridad en los cuidados paliativos, ya que está directamente relacionado con la promoción del confort y la calidad de vida^(2)^. En este contexto, la medición sistemática de la intensidad de los síntomas se vuelve esencial para orientar intervenciones individualizadas y evaluar la efectividad de las prácticas de enfermería orientadas al alivio y al bienestar^(3)^.

El confort físico representa la experiencia de alivio y tranquilidad derivada de la satisfacción de las necesidades corporales básicas y del control del dolor y de otros síntomas^(1,2,3)^. La ausencia de confort se traduce en malestar, fatiga y sufrimiento, lo que interfiere en el equilibrio homeostático y emocional del paciente con cáncer^(4)^. Por lo tanto, la evaluación del confort debe integrarse en la rutina asistencial, lo que permite identificar de manera temprana las alteraciones que impactan negativamente el estado general y la respuesta terapéutica al tratamiento^(5)^.

Estudios recientes con pacientes en cuidados paliativos señalan una correlación significativa entre los síntomas físicos intensos y los bajos niveles de bienestar general, lo que sugiere que el malestar es una consecuencia directa de la acumulación de síntomas^(3)^. La literatura evidencia que cuanto mayor es la intensidad del dolor y la fatiga, menores son las puntuaciones de bienestar físico y global^(4,5,6)^. Este hallazgo refuerza el papel del personal de enfermería en el monitoreo continuo de los síntomas, en la aplicación de escalas estandarizadas como la *Edmonton Symptom Assessment Scale (ESAS)* y en la adopción de prácticas basadas en la evidencia para el control de los síntomas^(7,8,9)^.

El uso del *Cuestionario de Confort General (Questionário de Conforto Geral-QCG)* en el contexto de los cuidados paliativos permite comprender el confort como un fenómeno multidimensional y evaluar de manera integrada sus dimensiones física, psicoespiritual, sociocultural y ambiental. En pacientes con cáncer hospitalizados, el QCG ha demostrado sensibilidad para detectar variaciones en el bienestar ante las intervenciones de enfermería, lo que permite identificar los factores que más comprometen el confort^(3,4,5)^. Esta herramienta ayuda a formular planes de atención centrados en las necesidades del paciente, especialmente en el control de los síntomas que afectan al cuerpo y al espíritu simultáneamente^(3,4,5,6,7)^.

De este modo, investigar la relación entre los síntomas físicos y el confort general contribuye no solo a la mejora de la práctica asistencial, sino también al fortalecimiento de los indicadores de calidad en cuidados paliativos. Al evidenciar que el malestar físico está fuertemente asociado a la intensidad sintomática, es posible orientar los esfuerzos interdisciplinarios hacia intervenciones tempranas y eficaces^(8,9,10,11,12)^. La integración entre la evaluación de los síntomas y la medición del bienestar debe considerarse un componente esencial de las buenas prácticas en enfermería paliativa, con un impacto directo en la dignidad, el bienestar y la humanización de la atención^(13)^.

Aunque la relación entre los síntomas físicos y el bienestar en pacientes con cáncer ya se reconoce en la literatura, se observa una escasez de estudios que investiguen estas asociaciones de manera correlacional en individuos hospitalizados en cuidados paliativos, especialmente en el contexto brasileño y utilizando instrumentos validados de manera combinada. La mayoría de las investigaciones se concentran en contextos ambulatorios o en fases específicas de la enfermedad^(7,9,14)^. Por lo tanto, el presente estudio tuvo como objetivo analizar las correlaciones entre la intensidad de los síntomas físicos y los niveles de confort general en pacientes con cáncer hospitalizados en cuidados paliativos.

## MÉTODO

### Tipo de Estudio

Se trata de un estudio cuantitativo, observacional, descriptivo y analítico, con diseño transversal, basado en la Teoría del Confort de Kolcaba^(1)^.

El diseño transversal permitió la recolección de datos en un único momento de la hospitalización, lo que favoreció el análisis de las interrelaciones entre los síntomas evaluados por la *ESAS* ^(3,15,16,17,18)^ y los niveles de confort medidos por el QCG^(1,11,12)^.

Los análisis de la asociación entre el confort general y las variables sociodemográficas se realizaron mediante pruebas de comparación de medias (prueba t de Student o análisis de varianza – ANOVA, según el tipo de variable), mientras que las relaciones entre la intensidad de los síntomas y los niveles de confort general se examinaron mediante el coeficiente de correlación de Pearson.

### Lugar de la Investigación

El estudio se llevó a cabo en el Hospital del Cáncer IV (HC IV) del Instituto Nacional del Cáncer (INCA), ubicado en la ciudad de Río de Janeiro, Brasil. El INCA es una institución vinculada al Ministerio de Salud y constituye el principal centro público de referencia nacional en prevención y control del cáncer, con cuatro unidades asistenciales distribuidas en la ciudad: HC I, HC II, HC III y HC IV.

El HC IV es la unidad de cuidados paliativos del INCA, destinada a la atención integral de pacientes con cáncer avanzado, sin posibilidad de curación, derivados de las demás unidades del Instituto. Su misión institucional es «promover y proporcionar cuidados paliativos oncológicos de la más alta calidad, con competencia técnica y humanitaria», centrándose en lograr la mejor calidad de vida posible para los pacientes y sus familiares mediante el control de los síntomas físicos, psicológicos, sociales y espirituales.

La unidad cuenta con una estructura multiprofesional completa, con servicios en las modalidades ambulatoria, domiciliaria, hospitalaria y de urgencias. El área de hospitalización, donde se llevó a cabo la recolección de datos, consta de cuatro pisos, cada uno con 14 camas distribuidas en habitaciones dobles e individuales, lo que suma un total de 56 camas destinadas al control de síntomas, la realización de procedimientos quirúrgicos electivos y los cuidados al final de la vida.

### Participantes de la Investigación

La población del estudio estuvo compuesta por pacientes con cáncer hospitalizados en el Hospital del Cáncer IV (HC IV) del Instituto Nacional del Cáncer (INCA), en cuidados paliativos, durante el período de Recolección establecido en la investigación. Se incluyeron pacientes adultos, de ambos sexos, con edad igual o superior a 18 años, diagnosticados con cáncer avanzado y en fase no sensible al tratamiento modificador de la enfermedad, según el registro en la historia clínica. Los criterios de inclusión abarcaron a pacientes con condiciones clínicas y cognitivas preservadas para responder a los instrumentos de investigación, con un tiempo mínimo de hospitalización de 24 horas y que aceptaron participar voluntariamente mediante la firma del Término de Consentimiento Libre e Informado (TCLE).

Se excluyó a los pacientes en sedación paliativa, con cuadro clínico inestable, deterioro cognitivo significativo o que presentaran limitaciones neurológicas que impidieran la comprensión de los ítems de los cuestionarios.

Para el cálculo de la muestra, se consideró un nivel de confianza del 90 % y un error muestral del 5 %. Debido a las características de la población investigada, se adoptó la proporción de 80/20 para una población homogénea, compuesta por pacientes con cáncer en cuidados paliativos. Se utilizó como referencia el número de ingresos en la unidad en el año 2023 (n = 1.351) y la tasa de ocupación del 71 %, lo que dio como resultado una muestra de 155 pacientes.

Los datos se obtuvieron mediante la aplicación de cuestionarios estructurados y escalas realizadas a pie de cama por la investigadora responsable y por becarios capacitados, quienes siguieron un protocolo estandarizado de abordaje y recolección. El tiempo promedio para la aplicación de los instrumentos fue de aproximadamente 25 minutos por participante, y los cuestionarios fueron completados conjuntamente por el investigador y el paciente, lo que garantizó una mejor comprensión de los ítems y minimizó los sesgos en las respuestas.

### Instrumentos de Recolección de Datos

La recolección de datos se llevó a cabo mediante la aplicación de un instrumento sociodemográfico y clínico estructurado, elaborado por la investigadora, y de dos instrumentos validados para la población brasileña: el QCG y el *ESAS*.

El instrumento sociodemográfico y clínico incluyó variables relacionadas con la caracterización de los participantes, como edad, sexo, creencia religiosa, ingreso mensual, tipo y estadio del cáncer, funcionalidad y presencia de dispositivos y lesiones.

Esta información se obtuvo mediante la aplicación de un cuestionario estructurado y se complementó con la consulta de los expedientes médicos, lo que garantizó la integridad y la fiabilidad de los datos.

Se utilizó el QCG para medir el nivel de confort de los pacientes. El instrumento se compone de 48 ítems distribuidos en cuatro dimensiones: física, psicoespiritual, sociocultural y ambiental, evaluadas en una escala de Likert de cuatro puntos (1 = totalmente en desacuerdo a 4 = totalmente de acuerdo). La puntuación total se calcula mediante la media aritmética de las respuestas, variando de 1 a 4, siendo los valores más altos indicativos de mayor confort. La versión brasileña presenta una excelente consistencia interna (α de Cronbach = 0,89), según estudios previos realizados en poblaciones hospitalarias y de cuidados paliativos^(17,18,19)^.

Se utilizó la *ESAS* para identificar la intensidad de los síntomas físicos y emocionales más comunes en pacientes con cáncer avanzado. La escala contiene nueve ítems que miden: dolor, fatiga, náuseas, depresión, ansiedad, somnolencia, apetito, bienestar y disnea, cada uno evaluado en una escala numérica de 0 a 10, en la que 0 representa la ausencia y 10 la máxima intensidad del síntoma^(20,21,22)^. La *ESAS* se utiliza ampliamente en la práctica clínica y en la investigación por su facilidad de aplicación, sensibilidad a las variaciones sintomáticas y validez transcultural, y es recomendada por la *European Association for Palliative Care* y la *American Society of Clinical Oncology* ^(22,23)^.

Ambos instrumentos se aplicaron en la misma sesión, en un ambiente tranquilo, durante el período diurno, respetando las condiciones clínicas y emocionales del paciente. La investigadora principal supervisó todas las etapas de la recolección de datos, garantizando la uniformidad en los procedimientos y minimizando los sesgos de aplicación.

## RESULTADOS

La caracterización sociodemográfica de la muestra reveló un perfil predominantemente femenino, con una edad media de 58 años, bajos ingresos mensuales y la mayoría declaró tener algún vínculo religioso. En cuanto a las condiciones clínicas, se observó un marcado deterioro funcional y una elevada frecuencia de uso de dispositivos médicos, hallazgos compatibles con el contexto de hospitalización en cuidados paliativos. También se constató diversidad en cuanto a los diagnósticos oncológicos y a las condiciones asociadas a la progresión de la enfermedad (Tabla 1).

**Tabla 1:** Características sociodemográficas y clínicas de los participantes (n = 155) – Río de Janeiro, RJ, Brasil, 2025.

Variables	n (%)
**Sexo**	
Mujer	106 (68,39)
Masculino	49 (31,61)
**Grupo de edad (años)**	
20 a 30	5 (3,23)
31 a 40	15 (9,68)
41 a 50	31 (20,00)
51 a 60	32 (20,65)
61 a 70	43 (28,29)
>70	26 (17,11)
**Tiene religión**	
Sí	139 (89,68)
No	16 (10,32)
**Ingresos mensuales (salario mínimo)**	
Sin ingresos	27 (17,42)
Hasta 1	86 (55,48)
De 1 a 2	29 (18,71)
3 a 4	11 (7,10)
>4	2 (1,29)
**Diagnóstico oncológico**	
Cánceres abdominales	45 (29,03)
Ginecológicos	36 (23,23)
De mama	23 (14,84)
Otros	51 (32,90)
**Funcionalidad (KPS)**	
KPS 30 %	93 (60,00)
KPS 40 %	32 (20,65)
KPS 50 %	25 (16,13)
KPS 60 %	5 (3,23)
**Dispositivos clínicos/lesiones**	
Estomas	37 (23,87)
Catéteres/drenajes	62 (40,00)
Acceso vascular/subcutáneo	149 (96,13)
Heridas tumorales	29 (18,60)
Lesión por presión	40 (25,81)

Leyenda: KPS = Karnofsky Performance Status.

La evaluación de los síntomas mediante la aplicación de la ESAS puso de manifiesto una elevada carga sintomática entre los pacientes con cáncer hospitalizados en cuidados paliativos. La ansiedad, la falta de apetito y la depresión presentaron las medias de intensidad más elevadas, seguidas de la somnolencia y la fatiga, tal y como se muestra en la Tabla 2.

**Tabla 2:** Caracterización de la escala ESAS (n = 155). – Río de Janeiro, RJ, Brasil, 2025.

Síntomas	Media (±DE)	Mediana (IQR)	Mínimo/Máximo
Dolor	3,60 (3,55)	3 (10)	0/10
Cansancio	4,10 (3,28)	4 (10)	0/10
Náuseas	2,48 (3,22)	0 (10)	0/10
Depresión	4,63 (3,68)	5 (10)	0/10
Ansiedad	5,51 (3,56)	7 (10)	0/10
Somnolencia	4,08 (3,53)	4 (10)	0/10
Falta de apetito	5,52 (3,42)	6 (10)	0/10
Disnea	1,35 (2,48)	0 (8)	0/9
Sensación de bienestar	6,40 (3,18)	7 (10)	0/10
Suma total	37,68 (17,30)	38 (67)	1/76

Leyenda: DE = desviación estándar.

Al correlacionar los síntomas evaluados por la escala ESAS con el QCG, se observó una correlación negativa estadísticamente significativa entre las puntuaciones de la escala ESAS y del QCG para la mayoría de los síntomas evaluados, con la excepción de la disnea. Este hallazgo pone de manifiesto que el aumento de la intensidad de los síntomas se asoció a una reducción de la puntuación en el QCG (Tabla 3).

**Tabla 3:** Correlación entre los síntomas de la escala ESAS y el QCG (n = 155) – Río de Janeiro, RJ, Brasil, 2025.

Escala de Evaluación de Síntomas	Cuestionario de Confort General	
	*r*	*p*
Dolor	-0,374	<0,001
Cansancio	-0,409	<0,001
Náuseas	-0,247	0,002
Depresión	-0,504	<0,001
Ansiedad	-0,448	<0,001
Somnolencia	-0,172	0,031
Falta de apetito	-0,327	<0,001
Disnea	-0,105	0,193
Sensación de bienestar	-0,535	<0,001
Suma total	-0,614	<0,001

Leyenda: *r* = coeficiente de correlación de Pearson; *p* = valor de significación estadística.

No se observaron asociaciones estadísticamente significativas entre las puntuaciones de bienestar y las variables sociodemográficas. Sin embargo, al analizar el bienestar en la dimensión psicoespiritual, se observó una diferencia estadísticamente significativa entre los participantes que declararon tener una religión y los que no la tenían (p = 0,045). Las personas con alguna creencia religiosa presentaron puntuaciones medias más elevadas de bienestar psicoespiritual (media: 51,28; DE: ± 7,65) en comparación con aquellas sin religión (media: 47,06; DE: ± 10,09), tal y como se muestra en la Tabla 4.

**Tabla 4:** Correlación entre el dominio psicoespiritual del QCG y las religiones de los participantes en el estudio (n = 155) – Río de Janeiro, RJ, Brasil, 2025.

Variables	Media (±DE)	Valor de p
**Tiene religión**		0,045^||^
Sí	51,28 (7,65)	
No	47,06 (10,09)	
**Religión**		0,090^‡^
Evangélica	51,10 (8,01)	
Católica	52,0 (7,23)	
Sin religión	47,06 (10,09)	
Espiritista	51,63 (5,14)	
Otras*	44,6 (10,62)	

Leyenda: ^||^Prueba t de Student;

^‡^ANOVA de un factor.

Al evaluar el bienestar de los pacientes por dominio del QCG, se observó que el dominio físico obtuvo las puntuaciones medias más bajas (media: 20,50; DE: ±6,24) y que los dominios psicoespiritual (media: 50,84; DE: ±8,0) y ambiental (media: 32,13; DE: ± 5,30) obtuvieron las medias más altas (Tabla 5).

**Tabla 5:** Caracterización de los dominios del bienestar según el QCG (n = 155) – Río de Janeiro, RJ, Brasil, 2025.

Ámbitos	Media (±DE)	Mediana	P25	P75	Mínimo/Máximo
Físico	20,50 (6,24)	20	10	34	10/36
Medio ambiente	32,13 (5,30)	32	22	42	15/43
Sociocultural	22,10 (4,60)	21	15	33	9/36
Psicoespiritual	50,84 (8,0)	51	30	67	25/69
Comodidad general	125,58 (18,52)	126	89	167	64/174

Leyenda: DE = desviación estándar.

## DISCUSIÓN

Los resultados de esta investigación revelan de manera consistente que el aumento de la intensidad de los síntomas físicos se asocia inversamente con el bienestar general y físico percibido por los pacientes con cáncer hospitalizados en cuidados paliativos. Esta correlación negativa, observada en síntomas como el dolor y la fatiga, confirma la hipótesis de que el malestar es una consecuencia directa de la acumulación de síntomas, lo que afecta no solo al bienestar físico, sino también al emocional y espiritual del paciente. A partir de esta constatación, se reafirma el supuesto de la Teoría del Bienestar de Kolcaba, según la cual el bienestar resulta de la satisfacción de las necesidades humanas en múltiples dimensiones, siendo la física la más sensible a los cambios derivados de la enfermedad avanzada^(16,17)^.

El hallazgo de que el dolor y la fatiga presentaron correlaciones negativas con el confort físico concuerda con estudios que identifican estos síntomas como los más incapacitantes y prevalentes en pacientes en cuidados paliativos^(18,19,20)^. En un análisis internacional que incluyó a más de 1,000 pacientes, los autores demostraron que el manejo inadecuado del dolor compromete la adherencia a las terapias y reduce la percepción de bienestar^(19)^. Del mismo modo, observaron que el control sintomático efectivo fue uno de los principales predictores de la satisfacción con la atención recibida, lo que indica que las intervenciones dirigidas al dolor y la fatiga tienen un impacto directo en la percepción del confort^(20)^. En cuanto a la disnea, aunque sin significación estadística en este estudio, se trata de un síntoma clínicamente relevante, especialmente por ser un síntoma angustiante asociado a la percepción de vulnerabilidad y al miedo a morir^(21)^.

Los estudios cualitativos señalan que la sensación de falta de aire tiende a exacerbar el sufrimiento psicológico y a aumentar la sensación de pérdida de control. Esta dimensión subjetiva refuerza la necesidad de enfoques integrados que asocien las terapias farmacológicas con el apoyo emocional y espiritual, ampliando el alcance de la atención más allá del control fisiológico del síntoma^(18,19,20,21,22)^. La relación entre el bienestar y los síntomas emocionales, como la ansiedad y la depresión, también se puso de manifiesto de manera significativa en los resultados. La correlación negativa observada indica que el sufrimiento emocional actúa como mediador del malestar general, interfiriendo en la forma en que el paciente experimenta los síntomas físicos y la propia hospitalización. Investigaciones recientes con la *ESAS*refuerzan que el sufrimiento psicológico influye fuertemente en el puntaje total de la escala, ampliando la percepción de fatiga y dolor^(19)^. Las intervenciones psicológicas y de comunicación empática promueven no solo una mejoría emocional, sino también un aumento del confort físico y social^(22)^.

Otro aspecto relevante es que la puntuación media de confort general observada en este estudio fue superior a la reportada en estudios internacionales con muestras similares, lo que sugiere la eficacia del modelo de atención adoptado por el Hospital del Cáncer IV/INCA. Esta unidad sigue el principio de la atención centrada en el paciente y la familia, con una fuerte integración multiprofesional, lo que se alinea con las recomendaciones de la *Organización Mundial de la Salud* y la *Sociedad Americana de Oncología Clínica* ^(23)^. La presencia constante de equipos de enfermería, medicina, nutrición, psicología, fisioterapia y capellanía favorece una atención integral, promoviendo tanto el alivio de los síntomas como el fortalecimiento emocional y espiritual del paciente.

En el estudio se observó que el dominio físico presentó la media más baja entre los dominios evaluados, lo que indica un mayor deterioro y refleja el impacto negativo de los múltiples síntomas físicos que experimentan los pacientes en cuidados paliativos.

La dimensión psicoespiritual, aunque no fue el foco principal de la correlación, se destacó entre las puntuaciones más altas del *QCG*. Este resultado corrobora las evidencias de que la espiritualidad actúa como mediadora positiva del confort, permitiendo resignificar el dolor y aceptar la finitud^(21,22)^. En una revisión reciente^(16)^, los autores demostraron que las intervenciones basadas en la Teoría del Confort de Kolcaba son eficaces para reducir la ansiedad y aumentar la sensación de paz interior, especialmente en contextos de fin de vida. Dichos hallazgos reiteran que el confort trasciende el alivio físico, siendo un fenómeno complejo que integra dimensiones existenciales, sociales y emocionales.

Desde el punto de vista metodológico, este estudio avanza en la comprensión empírica de la Teoría del Confort, al confirmar estadísticamente la interdependencia entre el malestar y la carga sintomática. La combinación de los instrumentos *ESAS* y *QCG* demostró ser sensible y complementaria para evaluar el estado clínico y subjetivo de los pacientes, lo que permitió identificar con precisión las áreas de mayor vulnerabilidad. Estudios recientes refuerzan el valor de los instrumentos estandarizados para el monitoreo del confort y del sufrimiento total, recomendando su uso rutinario en la práctica asistencial^(20,21,22)^.

Entre las limitaciones del estudio, se destacan el diseño transversal, que impide inferencias de causalidad, y el uso de una muestra no probabilística, restringida a una sola institución. A pesar de ello, la homogeneidad de la muestra y la estandarización de la Recolección de datos confieren solidez interna a los resultados. Como avance para la enfermería, los hallazgos reafirman la importancia de incorporar la medición del bienestar como indicador de calidad asistencial, además de contribuir a la elaboración de protocolos de cuidados basados en la evidencia. La integración entre la evaluación de los síntomas y el bienestar puede orientar las decisiones clínicas, promover el alivio del sufrimiento y fortalecer la humanización de los cuidados paliativos^(19,21,22,23)^.

Los resultados de este estudio presentan implicaciones directas y significativas para la práctica de la enfermería en cuidados paliativos oncológicos, al evidenciar que la intensidad de los síntomas físicos y emocionales está fuertemente asociada a la disminución del confort percibido por los pacientes. Esta constatación refuerza la necesidad de que los enfermeros adopten el confort como indicador clínico de la calidad de la atención, integrando su evaluación en el proceso de enfermería, especialmente en las etapas de diagnóstico y planificación de la atención.

El uso conjunto de la ESAS y el QCG constituye una estrategia práctica, rápida y validada para el monitoreo continuo del bienestar del paciente. La aplicación sistemática de estos instrumentos puede orientar la priorización de las intervenciones de enfermería, promover el manejo oportuno de los síntomas y respaldar la toma de decisiones clínicas basadas en la evidencia. Desde el punto de vista ético y humanístico, los hallazgos reafirman que los profesionales de enfermería deben reconocer el confort como un derecho humano esencial, sobre todo en situaciones de terminalidad. El enfoque sensible, empático y centrado en la persona amplía la eficacia de las acciones paliativas y fortalece el vínculo terapéutico, favoreciendo la dignidad y la autonomía del paciente hasta el final de la vida.

Además, el estudio contribuye al perfeccionamiento de las prácticas de educación permanente en salud, al demostrar que la medición del confort puede incorporarse como competencia esencial en los protocolos de atención y en las rutinas asistenciales. La formación continua de los equipos de enfermería, centrada en la evaluación integrada de los síntomas y el confort, potencia la calidad y la seguridad de la atención prestada en instituciones de salud públicas y privadas.

## CONCLUSIÓN

El estudio evidenció que el aumento de la intensidad de los síntomas físicos reduce significativamente el confort general de los pacientes con cáncer hospitalizados en cuidados paliativos. Estos hallazgos confirman que el manejo efectivo de los síntomas físicos constituye un elemento central para el mantenimiento del bienestar y la dignidad en el proceso de morir, además de reforzar que el confort no es solo un resultado deseable de la atención, sino un indicador clínico sensible a la calidad de la asistencia prestada.

Los resultados refuerzan la importancia del equipo de enfermería, en el contexto interdisciplinario, en la implementación de estrategias terapéuticas que combinan competencia técnica, sensibilidad ética y atención humanizada. La incorporación sistemática de la evaluación del bienestar y de los síntomas en las rutinas asistenciales permite la identificación temprana de complicaciones y la planificación de intervenciones individualizadas, con un seguimiento continuo de los resultados.

De esta manera, la investigación contribuye a consolidar el confort como un parámetro medible de la calidad de la atención de enfermería brindada a pacientes con cáncer en cuidados paliativos.

## Data Availability

Todo el conjunto de datos que respalda los resultados de este estudio se publicó en el propio artículo.
